# A Continuous Renal Replacement Therapy Protocol for Patients with Acute Kidney Injury in Intensive Care Unit with COVID-19

**DOI:** 10.3390/jcm9051529

**Published:** 2020-05-19

**Authors:** Federico Nalesso, Francesco Garzotto, Leda Cattarin, Laura Gobbi, Laila Qassim, Luca Sgarabotto, Ivo Tiberio, Lorenzo A. Calò

**Affiliations:** 1 Department of Medicine, Nephrology, Dialysis and Transplantation Unit, University of Padova, 35128 Padova, Italy; federico.nalesso@aopd.veneto.it (F.N.); leda.cattarin@gmail.com (L.C.); laura.gobbi@hotmail.it (L.G.); laila.87@virgilio.it (L.Q.); sgarabottoluca@gmail.com (L.S.); 2 Veneto Institute of Oncology IOV-IRCCS, Healthcare Directorate Unit, 35128 Padova, Italy; f.garzotto@gmail.com; 3 Emergency-Urgency Department, Anesthesia and Intensive Care, Azienda Ospedaliera di Padova University of Padova, 35128 Padova, Italy; ivo.tiberio@aopd.veneto.it

**Keywords:** Covid-19, AKI, ARDS, CRRT, CVVHD, HCO, cytokines, intensive care

## Abstract

COVID-19 often leads to acute respiratory distress syndrome complicated by acute kidney injury (AKI). The indications for renal replacement therapy for these patients are those commonly accepted to treat AKI. We describe a continuous veno-venous haemodialysis (CVVHD) protocol for AKI, which aims to provide the best treatment according to the particular patient’s and medical personnels’ needs in biohazard settings with limited human and technological resources. We designed a CVVHD protocol with a high cut-off (HCO) filter in regional citrate anticoagulation (RCA). The HCO filter in diffusion determines the enhanced cytokines clearance with less filter clotting due to a lower filtration fraction. In our hospital, at the beginning of the pandemic outbreak, we treated seven COVID-19 patients with AKI stage 2 and 3 and recorded the circuit lifespan and the number of interventions on monitors. CVVHD in RCA appears to be safe, effective and easy to be performed in a biohazard scenario using lower blood flows and less bag changes with fluid savings, a biohazard reduction and sparing of resources. Although the data come from a very small cohort, our protocol seems related to a low mortality.

## 1. Introduction

The SARS-CoV-2 infection, termed coronavirus disease 2019 (COVID-19), appeared for the first time in Italy, among the European countries, with two simultaneous outbreaks in the Lombardy and Veneto regions. Currently, COVID-19 cases are registered in all Italian regions and the epidemic is spreading through Europe. Our Veneto Regional Hub Hospital for COVID-19 in Padua, has firstly faced an increasing number of patients admitted to the intensive care unit (ICU) due to severe illness. Patients affected by COVID-19 present a dysfunctional immune system with an uncontrolled immune response, leading to a “cytokine storm” associated with a severe lung injury [1], as described for SARS-CoV, in which was described an increased proinflammatory cytokines level associated with pulmonary inflammation and extensive lung damage [2]. Patients affected by COVID-19 requiring ICU admission have higher levels of cytokines, suggesting that the “cytokine storm” is associated with the disease severity [3].

Acute kidney injury (AKI) is a common complication that occurs in 50–70% of ICU patients and is associated with worse outcome, with mortality rates of nearly 50% [4].

Conventional COVID-19 therapy typically starts with resuscitative measures in ICU [5,6]. While an effective therapy for COVID-19 does not exist yet, the indications for renal replacement therapy (RRT) should be those widely accepted to AKI treatment, restoring the immune homeostasis, removing inflammatory mediators causing acute respiratory distress syndrome (ARDS) and preventing and correcting the fluid balance when diuretics are not effective. Fluid overload, in fact, is a known independent risk factor for ICU mortality [7] and leads to several complications such as pulmonary edema, further worsening the respiratory system. A recent Acute Dialysis Quality Initiative conference has extensively addressed the topic of patient selection and timing for continuous renal replacement therapy (CRRT) [8]. Conversely, data on patients with COVID-19 are still discordant due to the large practice variation on the management of this disease [9,10], mainly for the different local experience and resources. Moreover, in a biohazard setting, it is necessary to provide a simple and easy CRRT to determine the renal substitution and multiorgan support [11] by reducing the “cytokine storm” and control the fluid balance. In a biohazard scenario, it is necessary to improve technological and human resources by optimizing patient care and reducing the risk of medical staff contamination. CRRTs are characterized by high technological complexity and the need for supervision and maintenance can become critical in this particular ICU setting. The complexity of ARDS patients requires intensive care including the planned pronation from the supine position and vice versa more times per day. Moreover, the performance of central venous catheters (CVC) for extracorporeal circulation (EC) can be suboptimal, inducing reduced blood flows, as in the case for patients undergoing extracorporeal membrane oxygenation (ECMO) if the CRRT is performed using a CVC in a central vein. The blood flow in the EC, in fact, becomes critical as low flows correspond to a greater risk of circuit coagulation, less depurative clearances and greater filtration fractions (FF) in convective processes such as continuous veno-venous hemofiltration (CVVH) compared with diffusive processes such as continuous veno-venous hemodialysis (CVVHD). In addition, convective therapies generally require higher blood flows compared with the continuous veno-venous hemodialysis (CVVHD), resulting in recurrent alarms for an inadequate blood flow with consequent therapy downtimes and loss of a depurative dose. Regarding the purification characteristics necessary for ARDS patients with AKI, which includes the removal of cytokines, the replacement of the renal function and the maintenance of the fluid balance, we have set up an extracorporeal blood purification technique able to remove cytokines, replacing the kidney function using relatively lower blood flows and FF, ensuring the longest circuit lifespan with no therapy downtimes. Furthermore, we identified the best depurative technique requiring the lowest number of maintenance interventions for the fluid bags changing in order to reduce staff exposure to the COVID-19 patients and to potentially infected effluent fluids.

## 2. Experimental Section

At the very beginning of the outbreak in our region, we organized a team of intensivists, nephrologists and engineers aimed to define the most efficient strategy to provide a CRRT in the frame of limited human and technological resources as in a critical biohazard setting.

With the current knowledge on COVID-19 and according to our local resources, we designed a continuous veno-venous haemodialysis with a high cut-off membrane (HCO) in regional citrate anticoagulation (RCA) in the so-called RCA-HCO-CVVHD treatment. Heparin is also available for this treatment when RCA is contraindicated. No extra CRRT anticoagulants are used on concomitant ECMO patients. The use of a HCO filter in the diffusive modality with lower blood flows determines a reduced filtration fraction (FF), with a high clearance of small and medium molecules and an enhanced high molecular weight clearance for cytokines [12]. Our standard protocol for RCA-CVVHD requires a HCO filter (Septex, Baxter, IL, USA) with a blood flow (Q_B_) of 120–160 mL/min, a dialysate calcium free flow (Q_D_) of 30 mL/Kg/h and a 4% concentrated citrate solution (136 mmol/L) at the flow of 3.0 mmol/L balanced by calcium chloride infusion (680 mmol/L) in a patient’s central vein according to a specific monitor algorithm (Prismaflex, Baxter, IL, USA) (Table 1). According to our protocol, systemic and post-filter ionized calcium (iCa) must be controlled after the beginning of the treatment at 30 min, 2 h, 6 h and every 6 h. In case of a citrate dose or calcium compensation variation, an extra control after 2 h is required. The total blood calcium (Ca_TOT_) concentration should be evaluated in order to calculate the Ca_TOT_/iCa at least once daily to early identify a citrate accumulation detected by a ratio > 2.25 [13]. The citrate dose and the calcium compensation are set depending on the serum calcium values at the planned checks according to their specific algorithms reported in Table 2 and Table 3. Finally, although the collection of biological samples for the evaluation of patients’ cytokine levels upon our treatment protocol would have been necessary, it was intentionally not done due to obvious safeness reasons given the patients’ SARS-CoV-2 infection. However, based on the abovementioned characteristics of HCO used and supported by a large documentation regarding its effect on the reduction/clearance of cytokines, an effect on patients’ cytokine levels is implied.

In case of an ECMO patient, or if citrate and heparin are contraindicated, the Q_B_ can be increased to 160–200 mL/min to reduce the circuit’s coagulation, maintaining the same Q_D_ with a standard dialysate solution selected according to a patient’s ions and bicarbonate levels (Table 1). In order to customize the treatment and reduce the need for electrolyte replacement (which would increase the nurse’s workload), solutions with different concentrations of potassium (2 and 4 mmol/L), magnesium (0.5 and 0.6 mmol/L) and phosphorus (0 and 1.2 mmol/L) allow a flexible electrolytes management in all patients treated with heparin or without anticoagulation.

To achieve a daily depurative dose of 25 mL/kg/h according to KDIGO, a higher value, usually ranging from 25 to 30 mL/Kg/h, needs to be prescribed considering the potential downtimes due to the alarms and the bag changes. Indeed, we calculated a mean time from the empty bag alarm to the complete resolution of 15 min for any bag change due to the need for personal protective equipment (PPE) in this biohazard contest in a COVID-19 ICU. In the protocol, the depurative dose of 30 mL/kg/h is estimated to be suitable for compensating the downtimes of our clinical reality.

## 3. Results

Seven patients were treated with the RCA-HCO-CVVHD protocol until now (Table 4). The mean age was 68.1 ± 6.77 years, most male (85.7%) and overweight (mean weight was 91.0 ± 11.34 kg). At baseline, 85.7% had hypertension, 57.1% has a pre-existing chronic kidney disease and 42.9% had diabetes mellitus.

The application of our protocol in all seven patients allowed to reach 72 h of treatment for all 19 circuits used (mean circuits for patients 2.71 ± 0.76), without technical downtimes due to a central venous catheter malfunction or circuit/filter coagulation. Over 1300 h of CVVHD therapy (mean hours per patient 195.43 ± 5.43) were guaranteed, interrupted only by the bags change and scheduled circuit reset on expiry. The mean blood flow rate was 144.3 ± 11.34 mL/min and the mean dialysis dose was 2757.1 ± 320.71 mL/h, both calculated per total time. The patients’ prone ventilation did not cause changes in the blood flows inducing alarms or discontinuations of the treatment due to the reduced blood flows set. The extracorporeal circulation stability has considerably reduced the nurses’ interventions, decreasing the risk of contamination. No staff biohazard contamination due to CVVHD patient management was experienced. As a precaution, the effluent disposal was collected through a biohazard bin and not through the standard drainage to avoid further contact with potentially contaminated fluid.

The systemic calcium compensation algorithm of the CRRT monitor has shown to maintain the serum calcium in the physiological range in all patients. No patient had signs of citrate toxicity.

All patients obtained a CRRT steady state in the control of ions, hydration status and acid base balance with urea and creatinine in the physiological range. No episodes of metabolic acidosis or alkalosis were reported during the CRRT treatments.

Although the data come from a very small cohort, our protocol seems related to a low mortality rate (two patients of seven).

## 4. Discussion

Although convective therapies (CVVH) are technically the most indicated modality for the removal of large molecules such as cytokine and myoglobin, the disadvantage of pre- or post-dilution on the circuit management limits their use in a biohazard setting, where the bleeding risk, the request for low effluent volumes and minimal technical interventions on the CRRT monitors are essential for the biocontainment. In particular in CVVH, pre-infusion decreases the depurative efficacy due to the blood dilution, while the post-infusion promotes filter clotting due to the increased FF and filter hemoconcentration [14] promoting a premature circuit and blood losses, and higher workloads and costs-resources. In addition, the increased blood flows required in CVVH can result in a greater number of alarms for CVC malfunction with a consequent need for technical interventions by personnel. This element is important in relation to the patients’ pronation as potential CVC dysfunctions can occur at a higher blood flow.

The advantages of RCA-HCO-CVVHD compared with the standard CVVH modality deal with a minor effluent volume, fewer bag interventions, a lower FF with a higher filter and circuit lifespan (also in the event of contraindications for the heparin use) and less alarm for complications related to the Q_B_ due to a CVC malfunction.

For an 80 kg patient without net fluid removal and a Q_B_ of 140 mL/min with a hematocrit of 0.30, the pre-infusion in the CVVH modality has to be set to 3200 mL/h, considering the dilution factor, compared with 2200 mL/h of dialysate in CVVHD to achieve the same depurative dose of 25–30 mL/kg/h. This dose in the CVVHD modality allows personnel to provide 11 bag changes (5000 mL bag) compared with 16 in CVVH. Four fewer interventions in CVVHD mean a gain of 60 min nurse time per treatment per day, a saving of 20 L (four bags) per treatment per day and four PPE kits, as well as a decreased risk of a contamination. All these factors are very important in a scenario of reduced human and medical supplies.

The use of RCA-HCO-CVVHD therefore allows an effective renal purification in patients with ARDS and AKI in ICUs in a scenario of reduced human resources. Furthermore, although we have not measured patients’ cytokine levels upon our treatment protocol, in addition to the large available documentation regarding cytokine removal by the HCO used in our protocol [12], the recovery of 5 out of 7 patients from respiratory failure, the end of their need of invasive mechanical ventilation and even their discharge from the ICU are important indirect clinical proofs to be considered. These clinical characteristics, which followed our treatments, in fact, make highly likely the impact of our treatments on cytokines, by reducing/clearing their levels.

The RCA-HCO-CVVHD advantages compared with an equivalent dose in CVVH are a lower effluent volume, fewer bag interventions, lower FF with a higher filter and circuit lifespan and less alarms for complications related to the Q_B_ due to a CVC malfunction. The possibility of performing the treatment with lower blood flows has resulted in greater stability of the extracorporeal circulation and fewer alarms with reduced downtimes. This stability, obtained with the reduced blood flows used in the protocol, has been maintained also during and after the patient’s pronation and supination maneuvers. Moreover, the absence of coagulation of the extracorporeal circulation obtained by RCA has determined the achievement of 72 h of treatment for all the circuits used, decreasing the nurses’ interventions. The absence of circuit clotting avoided significant blood losses requiring blood transfusion. Moreover, the reduction of the technical interventions on the CRRT monitors due to the greater stability of the extracorporeal circulation and the absence of treatment interruptions for circuit coagulation have limited the nurses’ contacts with the patient only to the bag changes. This has reduced the workload and the number of personal protective equipment used for the CRRT management, saving resources. In our experience, no nurses were contaminated during the procedures related to the CRRT administration and management.

## 5. Conclusions

From the data obtained until now, the RCA-HCO-CVVHD appears to be safe, effective and easy to apply in a biohazard scenario with limited human resources. Further clinical application of this protocol will allow the collection of more data to confirm the clinical effectiveness of RCA-HCO-CVVHD.

Finally, depending on the desired purification strategy, the local skills and the availability, alternatives treatment can include [11] the use of a high molecular weight filter such as an EMIC2 (Fresenius Medical Care, Bad Homburg Germany), an adsorptive hemofiltration filter such as oXiris (Baxter, IL, USA), the adsorption characteristics of the Hemofeel (Toray Medical Co. Ltd., Tokyo, Japan) in CVVHD or the use of sorbents such as polymyxin B (Toray Medical Co. Ltd., Tokyo, Japan), CytoSorb (CytoSorbents Europe GmbH, Berlin, Germany), HA-330 (Jafron, China) and the Alteco LPS adsorber (Alteco Medical AB, Lund, Sweden).

## Figures and Tables

**Table 1:** Protocol parameters for the use of Septex with Prismaflex ^§^.

Treatment Parameters	RCA-CVVHD	Heparin-CVVHD	CVVHD without Anticoagulation	RCA-CVVHD: Treatment for 80 Kg Patient
Blood flow rate (mL/min)	120–160	160–200	160–200	140
Dialysis dose (mL/kg/h)	30 ^A^	30 ^B,C^	30 ^B,C^	30 ^A^ (2400 mL/h)
4% citrate flow (mL/h)	160-210	-	-	185
Calcium compensation (%)	100 ^*^	-	-	100 ^*^
Citrate dose (mmol/L)	3.0 ^#^	-	-	3.0 ^#^

Systemic, post-filter ionized calcium (iCa) and total blood calcium concentration (Ca_TOT_) are checked according to the RCA-CVVHD protocol. RCA: regional citrate anticoagulation *: initial value, then according to the systemic iCa. #: initial value, then according to the post-filter iCa. A: dialysate calcium free (Prism0Cal B22^§^), B: emodiafiltration fluid (Prismasol 2^§^), C: emodiafiltration fluid (Phoxilium^§^) §:(Baxter, IL, USA).

**Table 2:** Algorithm for the citrate dose management.

Treatment Parameters			
	LOW	NORMAL	HIGH
Post-filter ionized calcium (mmol/L)	<0.25	0.25–0.50	>0.50
Citrate dose (mmol/L)	2.7 mmol/L	3.0 mmol/L	3.3 mmol/L

**Table 3:** Algorithm for the calcium compensation management.

Treatment Parameters					
Systemic ionized calcium (mmol/L)	<0.8	0.8–0.9	1.0–1.2	1.2–1.35	>1.35
Calcium compensation	+30%	+20%	-	−10%	−20%

**Table 4:** Patients and treatments characteristics.

Patients Characteristics at Baseline (n = 7)	
**Age (years), Mean ± SD**	68.1 ± 6.77
Male, n (%)	6 (85.7%)
Chronic kidney disease, n (%)	4 (57.1%)
Diabetes mellitus, n (%)	3 (42.9%)
Arterial hypertension, n (%)	6 (85.7%)
Weight (Kg), mean ± SD	91.0 ± 11.34
AKI stage 2, n (%)	6 (85.7%)
AKI stage 3, n (%)	1 (14.3%)
C reactive protein (mg/L), mean ± SD	153.28 ± 118.61
**Treatment Characteristics (RCA-HCO-CVVHDF)**	
Blood flow rate (mL/min), mean ± SD	144.3 ± 11.34
Dialysis dose (mL/h), mean ± SD	2757.1 ± 320.71
Number of circuits for patients, mean ± SD	2.71 ± 0.76
Hours of treatment for patients, mean ± SD	195.43 ± 5.43
